# Correlation analysis and diagnostic performance of CT/MRI fat quantification parameters in metabolic dysfunction–associated steatotic liver disease

**DOI:** 10.3389/fmed.2026.1763013

**Published:** 2026-04-21

**Authors:** Wenwen Chen, Hua Chao, Tiechen Xiao, Luyao Qian, Jianfeng Zha, Ting Pan, Huijuan Rui, Jin Zhang

**Affiliations:** Department of Radiology, Changzhou Cancer Hospital, Changzhou, Jiangsu, China

**Keywords:** CT, diagnostic performance, fat quantification, metabolic dysfunction–associated steatotic liver disease (MASLD), MRI

## Abstract

**Introduction:**

Early quantitative diagnosis of metabolic dysfunction–associated steatotic liver disease (MASLD) is crucial for preventing disease progression. Fat analysis and calculation technique (FACT) MRI fat quantification has shown potential diagnostic value. This study aimed to compare the diagnostic performance of CT and MRI fat quantification techniques and to evaluate their correlation with clinical and biochemical markers in in patients with MASLD.

**Methods:**

A retrospective analysis was conducted in 240 MASLD patients and 89 controls from December 2021 to February 2025. Demographic and clinical data, serum markers of liver function, inflammation, and lipid metabolism, and CT/MRI fat quantitative parameters were compared between groups. Correlations between CT/MRI parameters and serum markers were analyzed. All 329 participants were randomly divided into a training cohort (*n* = 230) and a validation cohort (*n* = 99) for diagnostic performance evaluation. Diagnostic performance was evaluated using ROC curves and AUC values, with the DeLong tests used for pairwise comparisons.

**Results:**

MASLD patients showed higher levels of transaminases, glutamyl transpeptidase, interleukins, total cholesterol, and triglycerides (all *P* < 0.05). MRI-derived fat fraction (FF) and R2^*^, as well as CT-derived fat concentration and the liver-to-spleen CT ratio, were significantly elevated (*P* < 0.05). FF showed the highest diagnostic performance (AUC), followed by R2^*^. CT parameters demonstrated acceptable but lower diagnostic performance diagnostic performance than MRI parameters.

**Conclusion:**

Given the exploratory nature of the study and the lack of uniform gold standard validation, the diagnostic performance findings of FACT-derived fat parameters (FF and R2^*^) should be interpreted with caution. FACT-derived fat parameters showed significant correlations with CT-based measures and serum markers and demonstrated higher diagnostic performance compared to CT-based parameters. These findings suggest FACT-derived MRI parameters may serve as useful non-invasive tools for assessing hepatic fat content in MASLD. However, further validation with accurate reference standards, such as MRI-PDFF or liver biopsy, is required to confirm their clinical utility.

## 1 Introduction

Metabolic dysfunction–associated steatotic liver disease (MASLD) is a common chronic liver disease characterized by excessive lipid accumulation in hepatocytes. Its incidence has increased steadily in recent years and deserves substantial clinical attention (1, 2). Disease onset is often insidious, and most patients remaining asymptomatic or showing no overt signs of liver dysfunction. Only a minority present with non-specific symptoms such as fatigue or abdominal pain (3). The pathogenesis of MASLD is multifactorial and involves genetic susceptibility, environmental influences, and complex intracellular and extracellular mechanisms. MASLD is a progressive disease with a potentially reversible early stage. If left uncontrolled, it can progress to cirrhosis, liver failure, and even liver cancer, thereby seriously affecting patient health (4). Therefore, early quantitative diagnosis and timely clinical intervention are crucial for preventing disease progression and have become important priorities in clinical practice.

MASLD is now considered the hepatic manifestation of metabolic syndrome and is closely associated with obesity, type 2 diabetes, insulin resistance, and dyslipidemia. Its global prevalence is estimated to exceed 25% and continues to rise with the growing burden of obesity (1, 2). While liver biopsy remains the gold standard for diagnosis, it is invasive, costly, and prone to sampling error, making it unsuitable for widespread use (5). These limitations highlight the need for accurate, reproducible, and non-invasive imaging-based diagnostic methods.

Current diagnostic methods for MASLD include serologic testing, liver biopsy, ultrasound, CT, and MRI (5). However, studies have shown that blood lipid levels are not directly proportional to hepatic fat content, and serologic and biochemical markers lack sufficient accuracy for diagnosing or predicting MASLD (6, 7). Ultrasound is convenient and widely used, but its sensitivity for detecting mild steatosis is limited (8–10). CT offers fast acquisition and is widely available, but its diagnostic performance can be compromised by iron deposition and beam-hardening artifacts, especially in patients with early disease (11, 12). In contrast, magnetic resonance imaging (MRI) allows for accurate, reproducible quantification of hepatic fat content and is less affected by confounding factors (13, 14). Ultrasound is convenient and widely used but has limited sensitivity for detecting mild steatosis (15). More recently, quantitative ultrasound techniques, including the controlled attenuation parameter (CAP), attenuation coefficient (AC), and backscatter coefficient (BSC), have been developed to improve the non-invasive assessment of hepatic steatosis. Although these methods correlate better with histologic fat grade than conventional B-mode ultrasound, they remain operator-dependent and may be less accurate in obese patients or in those with heterogeneous fat distribution (11, 12).

In recent years, the fat analysis and calculation technique (FACT) MRI sequence has emerged as a promising tool for hepatic fat quantification. It enables calculation of both fat fraction (FF) and R2^*^, the latter of which also provides information on iron deposition. FACT offers a reliable, non-invasive method to characterize hepatic steatosis and is gaining clinical interest (16). The FACT sequence operates with specific technical specifications, including echo times (TE) ranging from 1.45 ms to 3.27 ms, repetition times (TR) between 4.06 ms and 10.78 ms, and a field strength of 1.5T or 3T. These technical parameters contribute to its ability to quantify fat and iron content with high precision. However, its comparative diagnostic performance against traditional CT parameters and its association with biochemical markers in MASLD patients remain underexplored. The magnetic resonance FACT sequence enables non-invasive quantification of hepatic fat and iron deposition by generating fat fraction (FF) and R2^*^maps (16). Although FACT is a vendor-specific technology (United Imaging), its performance in liver-related imaging applications has shown promising results in some liver validation studies (17–19). However, at present, FACT technology is in the initial application in the diagnosis of MASLD, and while the diagnostic utility of FACT-derived FF has been well supported in previous studies (20), its correlation with liver function markers and inflammatory indices in MASLD patients, particularly in comparison to CT-based parameters, remains less well characterized. Recent studies have demonstrated the utility of FACT in providing comprehensive hepatic assessments, particularly in steatosis and iron overload, offering a reliable alternative when MRI-PDFF is unavailable (21, 22). Therefore, this study aimed to quantitatively assess the correlation between CT- and MRI-based fat quantification parameters particularly those derived from the FACT sequence and the diagnosis of MASLD. Additionally, we investigated their associations with liver function and lipid-related serum markers, and evaluated their diagnostic performance to provide evidence for the selection of non-invasive tools in clinical practice.

## 2 Method

### 2.1 General information

This retrospective diagnostic study included 329 participants with suspected MASLD from December 2021 to February 2025. Among these, 132 patients underwent liver biopsy, which served as the primary reference standard for validating CT- and MRI-derived fat quantification parameters. For the remaining participants (*n* = 197), hepatic steatosis was diagnosed non-invasively using liver ultrasound combined with MASLD clinical criteria (7), which served as the secondary reference standard for initial screening.

Ultrasound was selected as the non-invasive screening tool for hepatic steatosis because it is widely available, cost-effective, and routinely used in clinical practice. Although MRI-PDFF is considered a standard non-invasive technique for fat quantification, it was not used in this study because of logistical limitations, including limited availability at some sites and higher cost. Therefore, liver ultrasound was used as a practical alternative for screening hepatic steatosis, in accordance with international MASLD guidelines.

#### 2.1.1 Clarification of reference standard for ROC analysis

For clarity, all ROC analyses and AUC calculations evaluating the diagnostic performance of the imaging techniques were performed in the biopsy-confirmed subset of 132 patients. Histopathology served as the primary reference standard because it is the gold standard for grading hepatic steatosis. This approach was used to maximize the accuracy and interpretability of the diagnostic analyses.

For the remaining patients (*n* = 197), who were diagnosed non-invasively by ultrasound combined with clinical criteria, ultrasound served as the secondary reference standard for initial screening and was not used for the ROC analysis. These patients were included in descriptive and correlation analyses to explore the broader applicability of the imaging biomarkers.

#### 2.1.2 Clarification of validation cohort

In this study, ROC analyses were performed to assess the diagnostic performance of the imaging biomarkers, including FF, R2, and the liver-to-spleen ratio, for differentiating MASLD from controls. The validation cohort was an independent group used to test diagnostic performance and was not used to build a predictive model. ROC analyses were conducted to determine the AUC and diagnostic accuracy of these imaging biomarkers. Tables 1, 2 were updated to report the AUCs and 95% confidence intervals for the validation cohort, thereby improving clarity and transparency. The MASLD group was defined based on the international expert consensus criteria proposed in 2020 (7). Specifically, patients were included if they met both of the following conditions: (1) evidence of hepatic steatosis on liver ultrasound and (2) at least one metabolic risk factor, including overweight or obesity (body mass index [BMI] ≥ 25 kg/m^2^), type 2 diabetes mellitus, or metabolic dysregulation (e.g., elevated triglycerides, reduced high-density lipoprotein cholesterol [HDL-C], increased waist circumference, hypertension, or insulin resistance). All patients in the MASLD group met these criteria.

**Table 1:** Efficacy analysis of CT and MRI fat quantitative parameters in the training set for diagnosing MASLD.

Index	AUC	Youden Index	Cutoff value	Standard error	Specificity (%) (95% CI)	Sensitivity (%) (95% CI)	*95%CI for AUC*	*P-Value*
Energy spectrum CT fat concentration	0.855	0.781	332.80	0.028	90.40 (56/62)	87.60 (147/168)	0.801~0.909	< 0.001
Liver-spleen CT ratio	0.843	0.778	0.65	0.026	89.60 (55/62)	80.90 (136/168)	0.792~0.894	< 0.001
FF	0.989	0.775	6.61	0.006	98.30 (61/62)	100.00 (168/168)	0.976~1.000	< 0.001
R2^*^	0.946	0.924	17.51	0.020	97.47 (61/62)	93.30 (157/168)	0.907~0.985	< 0.001

AUC, area under the curve; CI, confidence interval; CT, computed tomography; FF, fat fraction.

**Table 2:** Verification of diagnostic efficacy of CT and MRI fat quantitative parameters in the validation set (*n* = 99).

Imaging parameter	Specificity (%) (95% CI)	Sensitivity (%) (95% CI)	PPV (%) (95% CI)	NPV (%) (95% CI)	Accuracy (%) (95% CI)	AUC (95% CI)	*P*-Value
Energy spectrum CT fat concentration	88.9% (24/27) [86.00-92.00]	85.3% (61/72) [81.00-89.00]	94.3% (61/65) [91.00-97.00]	73.2% (24/33) [65.00-80.00]	86.3% (85/99) [82.00-90.00]	0.855 (0.801–0.909)	< 0.001
Liver-spleen CT ratio	87.4% (23/27) [84.00–91.00]	79.3% (57/72) [74.00–84.00]	93.1% (57/61) [90.00–97.00]	69.6% (23/33) [61.00–77.00]	81.6% (81/99) [75.00–87.00]	0.843 (0.792–0.894)	< 0.001
Fat fraction (FF)	97.9% (26/27) [95.00–100.00]	98.7% (71/72) [96.00–100.00]	99.0% (71/72) [97.00–100.00]	97.1% (26/27) [93.00–100.00]	98.5% (97/99) [95.00–100.00]	0.989 (0.976–1.000)	< 0.001
R2^*^	96.4% (26/27) [93.00–100.00]	91.7% (66/72) [86.00–96.00]	97.8% (66/67) [94.00–100.00]	87.3% (26/32) [80.00–93.00]	93.0% (92/99) [88.00–97.00]	0.946 (0.907–0.985)	< 0.001

CT, computed tomography; PPV, positive predictive value; NPV, negative predictive value; FF, fat fraction.

**Figure 1:**
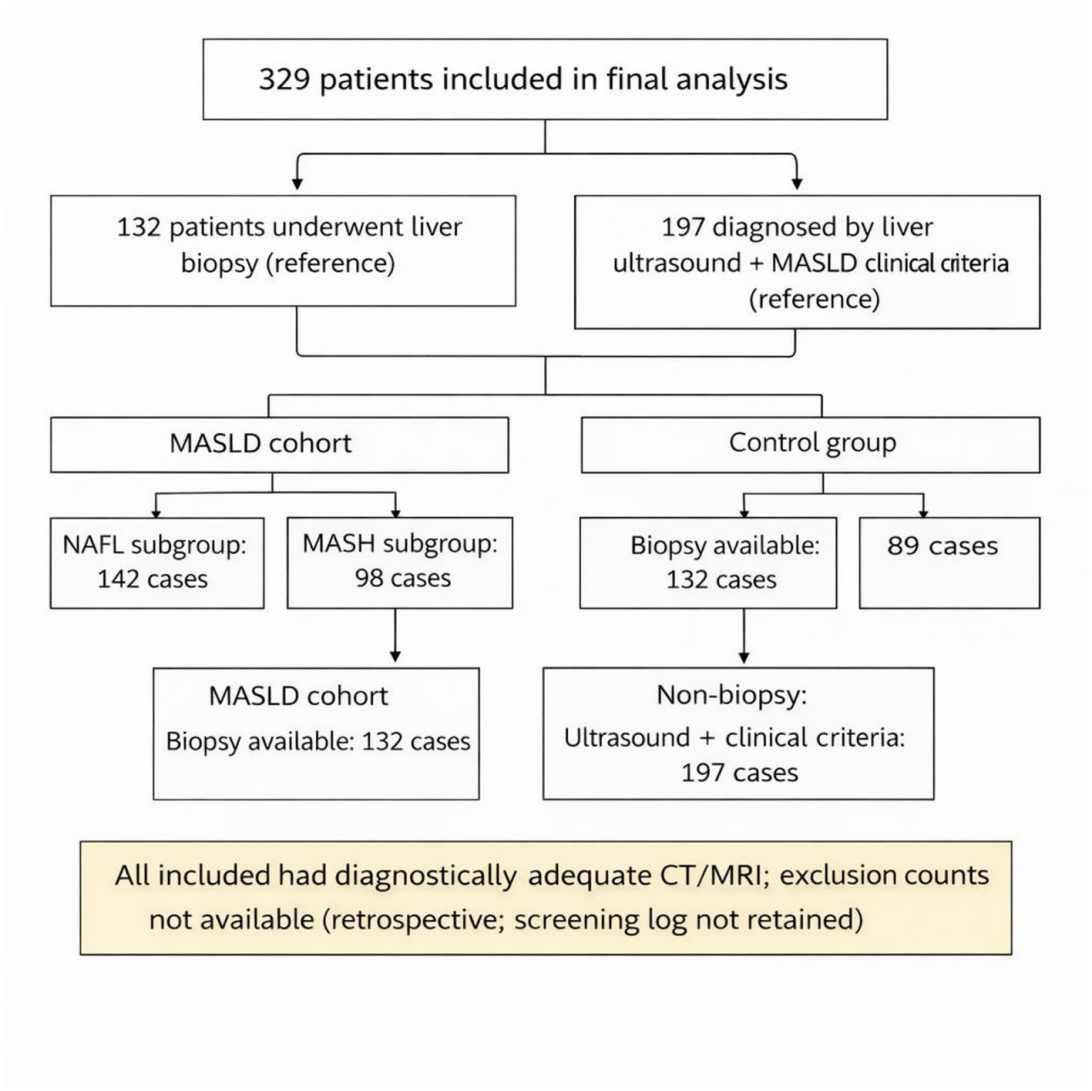
Flowchart showing patient enrollment and grouping. CT, computed tomography; MRI, magnetic resonance imaging; MASLD, metabolic dysfunction–associated steatotic liver disease; MASH, metabolic dysfunction–associated steatohepatitis.

A total of 329 patients were involved in the study, categorized according to histopathological findings from liver biopsy or ultrasound-based diagnosis combined with MASLD clinical criteria. The MASLD group comprised 240 patients (142 in the NAFL subgroup and 98 in the MASH subgroup), while the control group (CG) included 89 participants. Diagnostic performance analyses, including ROC and AUC evaluations, were conducted in the biopsy-confirmed subset of 132 patients to maximize accuracy and improve interpretability. The patient selection flow diagram is shown in Figure 1. The current study was approved by the Ethics Committee of the Changzhou Cancer Hospital. Due to the retrospective design and anonymized data, written informed consent was waived by the Ethics Committee.

The inclusion criteria were as follows: (1) age > 18 years; (2) for diagnostic performance analyses, only patients with biopsy-confirmed MASLD were included; (3) for descriptive and correlation analyses, participants with ultrasound-diagnosed MASLD (*n* = 197) were also included if they met the clinical diagnostic criteria for MASLD; (4) non-contrast CT and MRI, including the FACT sequence, performed within a 7-day interval; (5) no significant alcohol consumption (women < 70 g/week; men < 140 g/week); (6) controls were defined non-invasively and did not require liver biopsy unless clinically indicated; and (7) availability of complete demographic, clinical, and laboratory data, including anthropometric measurements (e.g., BMI and waist circumference), liver function tests (AST, ALT, γ-GT, ALP, total bilirubin, and albumin), lipid profile (TC, TG, HDL-C, and LDL-C), inflammatory markers (e.g., interleukins and TNF-α), and tumor markers (AFP and CEA), all obtained within seven days before CT and MRI to ensure temporal consistency. The control group (*n* = 89) was defined using non-invasive criteria and included participants with no evidence of hepatic steatosis on liver ultrasound, normal liver function tests, no personal or family history of chronic liver disease, and no metabolic dysfunction such as diabetes, dyslipidemia, or obesity. Liver biopsy was not routinely performed in controls unless clinically indicated.

Exclusion criteria include 1) presence of other liver diseases (e.g., drug-induced liver injury and viral hepatitis); 2) diseases affecting liver iron deposition (e.g., hereditary hemochromatosis); 3) absence of completed or diagnostically usable MRI (e.g., incomplete sequences or motion-degraded images); 4) CT examinations that did not meet predefined image-quality requirements for quantitative analysis (e.g., severe artifacts or incomplete data); 5) inability to cooperate with breath-holding during examination; 6) severe dysfunction of major organs such as heart and lung; 7) presence of liver tumors or other systemic diseases involving the liver; 8) history of liver or spleen surgery.

All participants included in the final analysis had CT and MRI examinations of sufficient quality for quantitative analysis. Because this was a retrospective study and a screening log of excluded examinations was not retained, the exact number of cases excluded because of unusable CT data or severe imaging artifacts could not be reliably reconstructed. Therefore, Figure 1 is presented as a cohort assembly diagram showing the final included cohort and subgroups rather than a complete screening flowchart with exclusion counts.

Although liver biopsy remains the gold standard for diagnosing and grading hepatic steatosis (23). t was not feasible for all participants in this retrospective imaging study because of its invasive nature. As a non-invasive alternative, hepatic steatosis was initially screened by ultrasound, and disease severity was inferred from quantitative imaging biomarkers, including CT-based fat concentration, the liver-to-spleen ratio, and MRI-based FF and R2^*^. These imaging parameters have been reported to correlate with steatosis grade, although histologic confirmation was not available for the entire cohort.

#### 2.1.3 Justification for focusing on CT and MRI

Advanced ultrasound techniques, including CAP, AC, and BSC, were not included as comparators in this study. We focused on CT and MRI because of their established clinical utility, availability at our institution, and documented value in assessing MASLD. In addition, the primary objective was to compare CT-based parameters with MRI fat quantification derived from FACT, which provides assessment of both hepatic fat content (FF) and iron deposition (R2). Because ultrasound-based techniques remain operator-dependent and may be less accurate in obese patients or in those with heterogeneous fat distribution, they were excluded to reduce bias and maintain methodological consistency.

### 2.2 General information collection

General demographic and clinical information for all enrolled subjects was gathered from the electronic system, including gender, age, marital status, waist circumference, and BMI. Serum indicators included γ-glutamyl transferase (γ-GT), alkaline phosphatase (ALP), aspartate aminotransferase (AST), total bilirubin, albumin, interleukins (IL-5, IL-1β, IL-6, IL-10, IL-2, IL-8, IL-4, IL-17, IL-12P70), α-interferon, γ-interferon, tumor necrosis factor (TNF), α-fetoprotein (AFP), carcinoembryonic antigen (CEA), triglyceride (TG), and total cholesterol (TC). All clinical and biochemical data were obtained within seven days prior to CT and MRI examinations to ensure temporal consistency and minimize variability in correlation analyses. The MASLD Activity Score (NAS) was calculated only for the subset of participants who underwent liver biopsy (*n* = 132) and was used exclusively for subgroup descriptive analyses, not for evaluating the diagnostic performance of imaging parameters. All serum biomarkers were obtained from our hospital's centralized electronic medical record system, which routinely archives laboratory data. Interleukin and cytokine panels are not measured for all patients by default but were available for this study because these markers are part of our hospital's standardized hepatology workup for patients with suspected steatotic liver disease. Only patients with complete biomarker data were included in the final analysis to ensure consistency across variables.

### 2.3 Energy spectrum CT examination

All participants fasted for at least 6 h before scanning and were positioned supine. Non-contrast dual-energy spectral CT was performed using a Canon Aquilion Prime CT scanner (Canon Medical Systems, Japan). The acquisition used rapid kilovoltage switching (100/140 kVp) with automatic tube current modulation (SureExposure 3D), matrix size 512 × 512, collimation 40 × 1.0 mm, rotation time 0.75 s, reconstructed slice thickness 3 mm, and interslice gap 3 mm. The scanning range extended from the top of the liver to the lower edge of the kidneys.

Using the vendor-provided GSI Volume Viewer software, hepatic fat concentration was calculated by spectral attenuation curve decomposition, which estimates lipid content based on material separation algorithms. In addition, conventional CT attenuation values (Hounsfield units) and the liver-to-spleen (L/S) attenuation ratio were derived for comparison.

ROI placement: To ensure reproducibility, three circular ROIs (1.5–2.0 cm^2^ each) were placed in homogeneous regions of the right hepatic lobe at segments V, VI, and VII, while avoiding visible vessels, bile ducts, focal lesions, and imaging artifacts. Three ROIs were also placed in the splenic parenchyma to calculate the L/S ratio. The right hepatic lobe was selected because it provides a larger and more homogeneous parenchymal area and is less affected by artifacts from the stomach, heart, and adjacent bowel gas than the left lobe. This approach is widely used in quantitative hepatic fat studies to reduce variability and measurement error.

Restricting ROI sampling to the right hepatic lobe improves consistency and reduces variabilityin patients with heterogeneous steatosis, in line with previous recommendations.

All measurements were performed independently by two experienced abdominal radiologists (≥5 years of experience), and any discrepancies were resolved by consensus.

### 2.4 FACT sequential fat quantification technology

**Figure 2:**
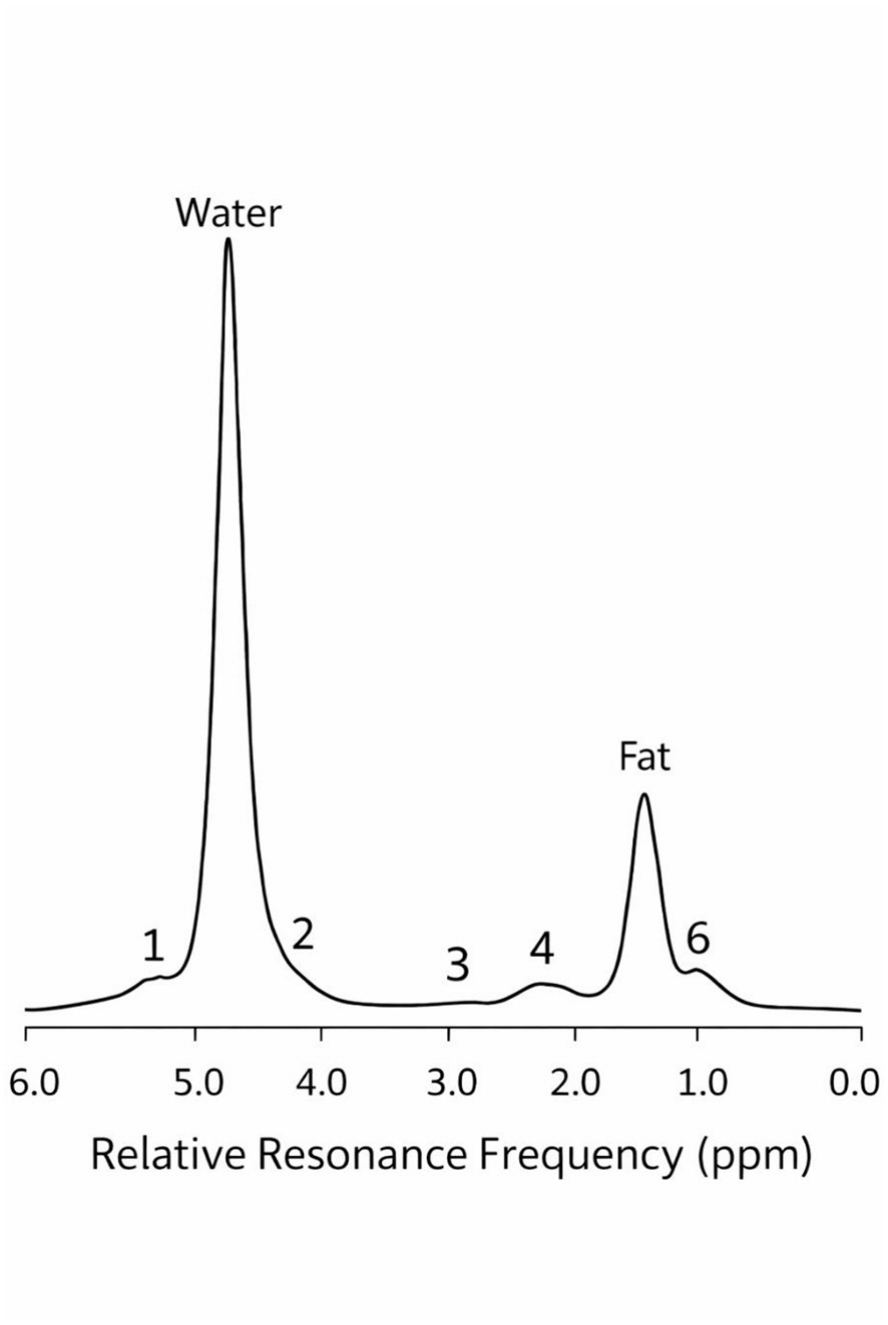
Conceptual illustration of the chemical shift difference between water and fat protons, which underlies Dixon-based MRI techniques such as the FACT sequence. This known frequency difference is utilized for water-fat separation but is corrected in post-processing.


MRI was performed using a uMR 780 system (Shanghai United Imaging Medical Technology Co., Ltd.). The FACT sequence is based on the chemical shift difference between water and fat protons, which enables accurate water–fat separation; the corresponding frequency spectrum is illustrated in Figure 2. The FACT sequence has been widely used in studies assessing fat quantification and liver health, as demonstrated in recent literature (24–26). All participants fasted for at least 6 h before the examination and underwent breath-hold training to reduce motion artifacts. Scanning was performed in the supine position using a multi-sequence FACT protocol designed to quantify both fat fraction (FF) and R2^*^. Five sequences were acquired under the FACT protocol, and the acquisition parameters were explicitly linked to their respective targets: TR = 10.78 ms, TE = 1.72 ms, flip angle = 3°, bandwidth = 900 Hz/pixel, slice thickness = 5.0 mm, acquisition time = 17 s for water-fat separation; TR = 4.06 ms, TE = 1.45 ms, flip angle = 10°, bandwidth = 800 Hz/pixel, slice thickness = 4.5 mm, acquisition time = 14 s for FF quantification; TR = 3.27 ms, TE = 3.27 ms, flip angle = 10°, bandwidth = 650 Hz/pixel, slice thickness = 4.5 mm, acquisition time = 16 s for multi-echo R2^*^mapping; TR = 1,500 ms, TE = 90 ms, flip angle = 120°, bandwidth = 800 Hz/pixel, slice thickness = 6.0 mm, acquisition time = 28 s for reference mapping; and TR = 5,541 ms, TE = 74 ms, flip angle = 90°, bandwidth = 2,370 Hz/pixel, slice thickness = 6.0 mm, acquisition time = 2.2 s for iron quantification.^*^ ROIs were manually defined on FF and R2^*^ maps by two experienced radiologists. Three circular ROIs (area: 1.5–2.0 cm^2^) were placed in the right hepatic lobe on three separate slices. The right hepatic lobe was chosen because it offers a larger, more homogeneous parenchymal area and is less affected by artifacts from the stomach, heart, and adjacent bowel gas compared to the left lobe. This approach is widely adopted in quantitative hepatic fat studies to minimize variability and measurement error. ROIs were placed carefully to avoid major vessels, bile ducts, calcifications, and imaging artifacts. High-quality imaging was objectively defined as images free from significant motion artifacts, field inhomogeneity, or slice misregistration, and scans not meeting these criteria were excluded. FF and R2^*^ maps were automatically generated using the United Imaging uWS-MR workstation (version SW 001.003, R003.1.67-2.473254-Re-20160918U3). Each ROI measurement was performed three times, and the average value was used for the final analysis.

$$\begin{array}{l} FF=\frac{\rho_{f}}{\rho_{w}+\rho_{f}}(27);R_{2}^{*}=\frac{1}{T_{2}^{*}}(28). \end{array}$$

The above CT and FACT imaging studies were reviewed by two board-certified radiologists with 8 years and 10 years of experience, respectively, in abdominal CT and MRI diagnosis. All ROI measurements were performed independently by the two radiologists, and both readers were blinded to clinical information, laboratory results, and histopathological findings during image analysis to minimize measurement bias. Interobserver reliability was assessed using the intraclass correlation coefficient (ICC) with a two-way random-effects model and absolute-agreement definition. ICC values with corresponding 95% confidence intervals (CIs) were calculated for each quantitative imaging parameter, including CT fat concentration, liver-to-spleen attenuation ratio, MRI fat fraction (FF), and R2^*^.

A consensus was reached after discussion, and in cases of disagreement, a third senior radiologist with 15 years of experience was consulted. If the two physicians could not agree on the diagnosis, a third physician with more than 5 years of experience in diagnosis was invited to discuss and reach a consensus.

### 2.5 Statistical analysis

To perform statistical analysis, SPSS 22.0 (IBM Corp.) was utilized. Continuous variables were tested for normality using the Shapiro–Wilk test. Normally distributed data are expressed as mean ± standard deviation (*x* ± s) and compared using independent-samples *t*-tests, whereas non-normally distributed data are reported as median (interquartile range) and compared using the Mann–Whitney U test. Cases and rates were used to convey count data. To compare groups, the chi-square test was employed. To evaluate the diagnostic performance of CT and MRI parameters in detecting MASLD, receiver operating characteristic (ROC) curves and the area under the curve (AUC) were calculated. To improve the robustness of diagnostic performance estimates and minimize potential overfitting, the entire dataset of 329 patients was randomly divided into a training cohort (*n* = 230; 168 MASLD, 62 CG) and an independent validation cohort (*n* = 99; 72 MASLD, 27 CG) in a 7:3 ratio. The training and validation cohorts included all 329 patients, encompassing both biopsy-confirmed (132 patients) and non-biopsy patients diagnosed via ultrasound and clinical criteria (197 patients). Optimal cut-off values for each imaging parameter were determined in the training cohort using the Youden index and subsequently applied unchanged to the validation cohort to calculate sensitivity, specificity, positive predictive value (PPV), negative predictive value (NPV), and overall accuracy. Pairwise comparisons of AUCs among different assessment tools were performed using the nonparametric DeLong test. Associations between imaging-derived parameters, including CT fat concentration, liver-spleen attenuation ratio, MRI fat fraction (FF), and R2^*^, and serum biomarkers, including AST, γ-GT, IL-6, IL-1β, IFN-γ, IL-8, IL-12p70, total cholesterol, and triglycerides, were analyzed using Pearson correlation for normally distributed variables and Spearman rank correlation for non-normally distributed variables. These analyses were intended to assess associations rather than diagnostic performance.

Interobserver reliability was assessed by calculating the intraclass correlation coefficient (ICC) for each quantitative parameter, including CT fat concentration, liver-spleen attenuation ratio, MRI fat fraction (FF), and R2^*^. ICCs were calculated from the two independent readings obtained before consensus using a two-way random-effects model with an absolute-agreement definition and average measures (ICC[2,k]), because the final estimates reflected the mean of the two readers. Ninety-five percent confidence intervals (95% CI) were obtained by bootstrapping with 1,000 resamples. ICC values were interpreted as follows: < 0.50, poor; 0.50–0.75, moderate; 0.75–0.90, good; and >0.90, excellent agreement. Bland-Altman plots were also generated to visualize bias and limits of agreement.

## 3 Results

### 3.1 General data comparison

There were no significant differences between the two groups in sex, age, marital status, waist circumference, BMI, or the following serum indicators: ALP, albumin, total bilirubin, interleukin-5, α-interferon, interleukin-2, interleukin-10, interleukin-17, interleukin-4, TNF, AFP, and CEA (all *P* > 0.05). However, AST, γ-GT, interleukin-6, interleukin-1β, γ-interferon, interleukin-8, interleukin-12P70, TC, and TG levels were significantly higher in the MASLD group than in the control group (all *P* < 0.05, Table 3). In the MASLD group, the mean NAS score was 2.31 ± 0.47 in the NAFL subgroup (*n* = 142) and 5.87 ± 0.63 in the MASH subgroup (*n* = 98) (Table 3).

**Table 3:** Comparison of general information.

Items	MASLD group (*n* = 240)	Control group (*n* = 89)	*χ^2^/t* value	*P* value
Gender			1.953	0.162
Male	150 (62.50)	63 (70.79)		
Female	90 (37.50)	26 (29.21)		
Age (years)	67.91 ± 5.46	66.88 ± 5.14	1.543	0.124
Marital status			2.067	0.151
Married	191 (79.58)	77 (86.52)		
Unmarried	49 (20.42)	12 (13.48)		
Waistline (cm)	91.72 ± 9.48	92.38 ± 9.67	0.558	0.577
BMI (kg/m^2^)	26.21 ± 2.17	25.93 ± 2.04	1.056	0.292
Aspartate aminotransferase (U/L)	43.59 ± 9.38	37.27 ± 5.47	5.987	< 0.001
Alkaline phosphatase (U/L)	110.40 ± 18.76	107.23 ± 17.24	1.391	0.165
Glutamyl transpeptidase (U/L)	57.34 ± 10.47	43.21 ± 7.46	11.675	< 0.001
albumin (g/L)	39.91 ± 6.75	38.74 ± 5.92	1.442	0.150
Total bilirubin (μmol/L)	14.41 ± 3.72	13.93 ± 3.47	1.058	0.291
Interleukin-5 (pg/mL)	4.25 ± 0.72	4.11 ± 0.67	1.596	0.112
alpha-interferon (IU/mL)	2.46 ± 0.63	2.39 ± 0.57	0.953	0.341
Interleukin-2 (U/L)	2.43 ± 0.60	2.29 ± 0.54	1.930	0.054
Interleukin-6 (pg/mL)	17.63 ± 2.37	5.38 ± 0.91	47.444	< 0.001
Interleukin-1β (pg/mL)	8.59 ± 1.95	4.72 ± 1.28	17.377	< 0.001
Interleukin-10 (pg/mL)	2.49 ± 0.76	2.37 ± 0.68	1.308	0.192
Interferon-gamma (pg/mL)	8.15 ± 1.53	7.38 ± 1.42	4.133	< 0.001
Interleukin-8 (μg/L)	39.53 ± 6.81	17.27 ± 2.31	30.174	< 0.001
Interleukin-17 (mIU/mL)	8.42 ± 2.29	7.94 ± 2.14	1.718	0.087
Interleukin-4 (pg/mL)	1.68 ± 0.31	1.64 ± 0.27	1.075	0.283
Interleukin-12P70 (pg/ml)	2.07 ± 0.45	1.79 ± 0.30	5.436	< 0.001
Tumor necrosis factor (μg/L)	3.09 ± 0.87	2.94 ± 0.75	1.439	0.151
AFP (ng/mL)	3.42 ± 1.15	3.28 ± 1.08	0.997	0.320
CEA (ng/mL)	2.87 ± 0.92	2.75 ± 0.88	1.063	0.289
TC (mmol/L)	6.32 ± 1.24	3.28 ± 1.18	20.009	< 0.001
TG (mmol/L)	2.37 ± 0.56	1.27 ± 0.25	17.869	< 0.001
NAS score				
MASLD (non-MASH) subgroup (*n* = 142)	2.31 ± 0.47			
MASH subgroup (*n* = 98)	5.87 ± 0.63			

BMI, body mass index; AFP, α-fetoprotein; CEA, carcinoembryonic antigen; TC, total cholesterol; TG, triglycerides; NAS, non-alcoholic fatty liver disease activity score; MASLD, metabolic dysfunction–associated steatotic liver disease; MASH, metabolic dysfunction–associated steatohepatitis.

### 3.2 Comparison of CT/MRI fat quantification parameters

**Figure 3:**
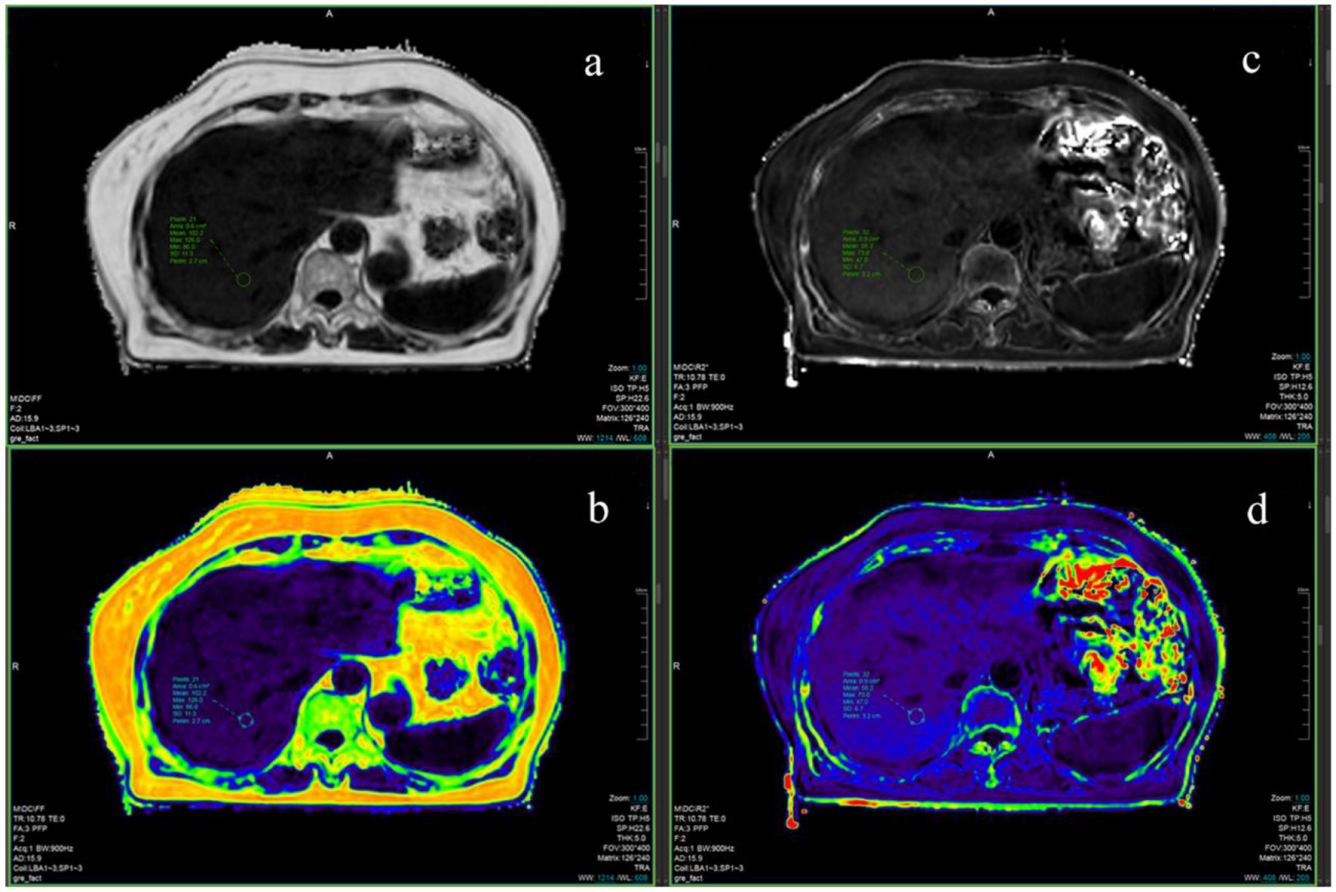
Pseudo-color map of FF and R2* calculation results of fat quantification [**(a)** FF TRA; **(b)** FF TRA; **(c)** R2* TRA; **(d)** R2* TRA].

Compared with the CG, it was found that the spectrum CT fat concentration, liver-to-spleen CT ratio, FF, and R2^*^ parameters of patients in the MASLD group were considerably increased (all *P* < 0.05, Table 4). Interobserver agreement for quantitative imaging measurements was excellent across all parameters. The ICCs (95% CI) were as follows: CT fat concentration, 0.94 (0.92–0.96); liver-spleen ratio, 0.91 (0.88–0.94); FF, 0.97 (0.95–0.98); and R2^*^, 0.95 (0.93–0.97). These ICCs were calculated from the two independent readings obtained before consensus adjudication. Bland-Altman analysis showed minimal bias and symmetrical limits of agreement. Representative pseudocolor maps of FF and R2^*^ are shown in Figure 3.

**Table 4:** Comparison of CT/MRI fat quantification parameters.

Group	Energy spectrum CT fat concentration	Liver-spleen CT ratio	FF	R2^*^
MASLD group (*n* = 240)	456.82 ± 23.34	1.02 ± 0.14	52.65 ± 4.58	67.79 ± 6.37
Control group (*n* = 89)	283.21 ± 22.85	0.47 ± 0.08	4.28 ± 0.85	12.71 ± 2.17
*t*	60.272	34.983	98.913	79.808
*P*	< 0.001	< 0.001	< 0.001	< 0.001

CT, computed tomography; FF, fat fraction.

### 3.3 Relationship between CT fat concentration and liver-spleen CT ratio and MRI fat quantitative parameters

**Figure 4:**
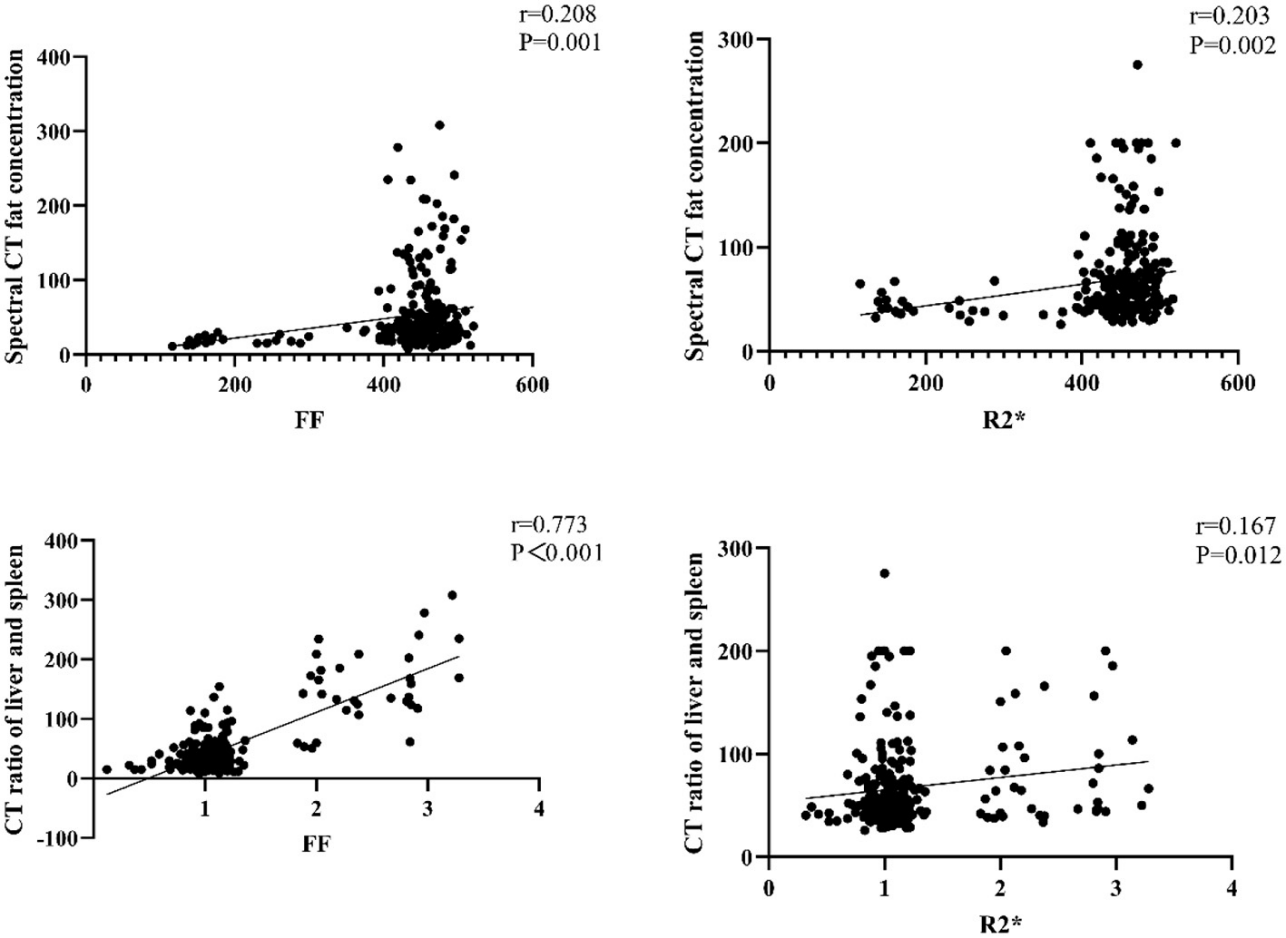
Correlation between CT (computed tomography), FF (fat fraction), and R2* in hepatic fat quantification.

Pearson correlation analysis showed that energy spectrum CT fat concentration was significantly correlated with both FF and R2^*^ (*P* < 0.05). Similarly, the liver-spleen CT ratio was significantly correlated with FF and R2^*^ (*P* < 0.05). These findings suggest that CT- and MRI-based quantitative parameters are associated with one another in the assessment of hepatic fat content in MASLD, as shown in Figure 4. However, in the absence of histologic confirmation or an MRI-PDFF reference standard, these correlations should be interpreted as preliminary associations between imaging biomarkers rather than definitive evidence of diagnostic performance.

### 3.4 Relationship between CT/MRI fat quantitative parameters and serum indexes

**Figure 5:**
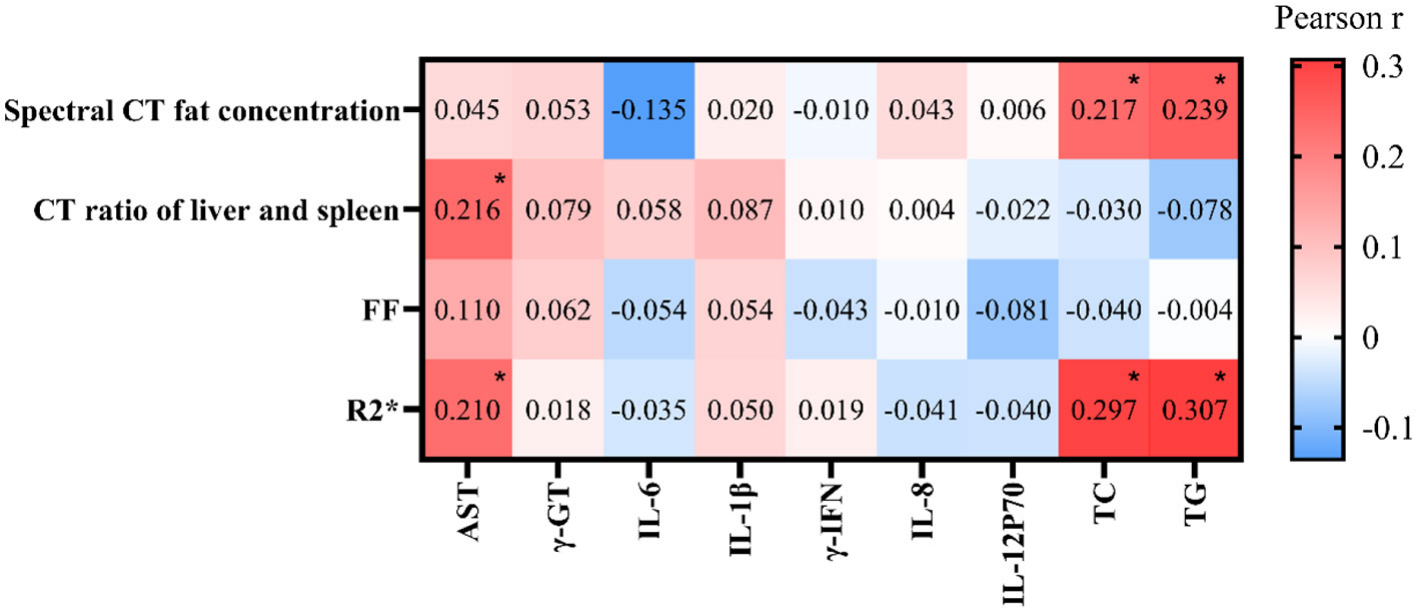
Correlation of CT (computed tomography) and FF (fat fraction) with serum biomarkers: AST (aspartate aminotransferase), γ-GT (γ-glutamyl transferase), IL (interleukins), TC (total cholesterol), and TG (triglycerides) in MASLD patients. Pearson correlation coefficients are shown.

Pearson correlation analysis revealed no significant correlations of energy spectrum CT fat concentration or FF with AST, γ-GT, IL-6, IL-1β, γ-IFN, IL-8, or IL-12P70 (*P* > 0.05). Similarly, the liver-spleen CT ratio and R2^*^ were not significantly correlated with γ-GT, IL-6, IL-1β, γ-IFN, IL-8, or IL-12P70 (*P* > 0.05). However, CT fat concentration and R2^*^ were positively correlated with AST (*P* < 0.05), and CT fat concentration, FF, and R2^*^ were positively correlated with TC and TG (*P* < 0.05), as shown in Figure 5.

### 3.5 Efficacy analysis of CT and MRI fat quantitative parameters in diagnosing non-alcoholic fatty liver disease

**Figure 6:**
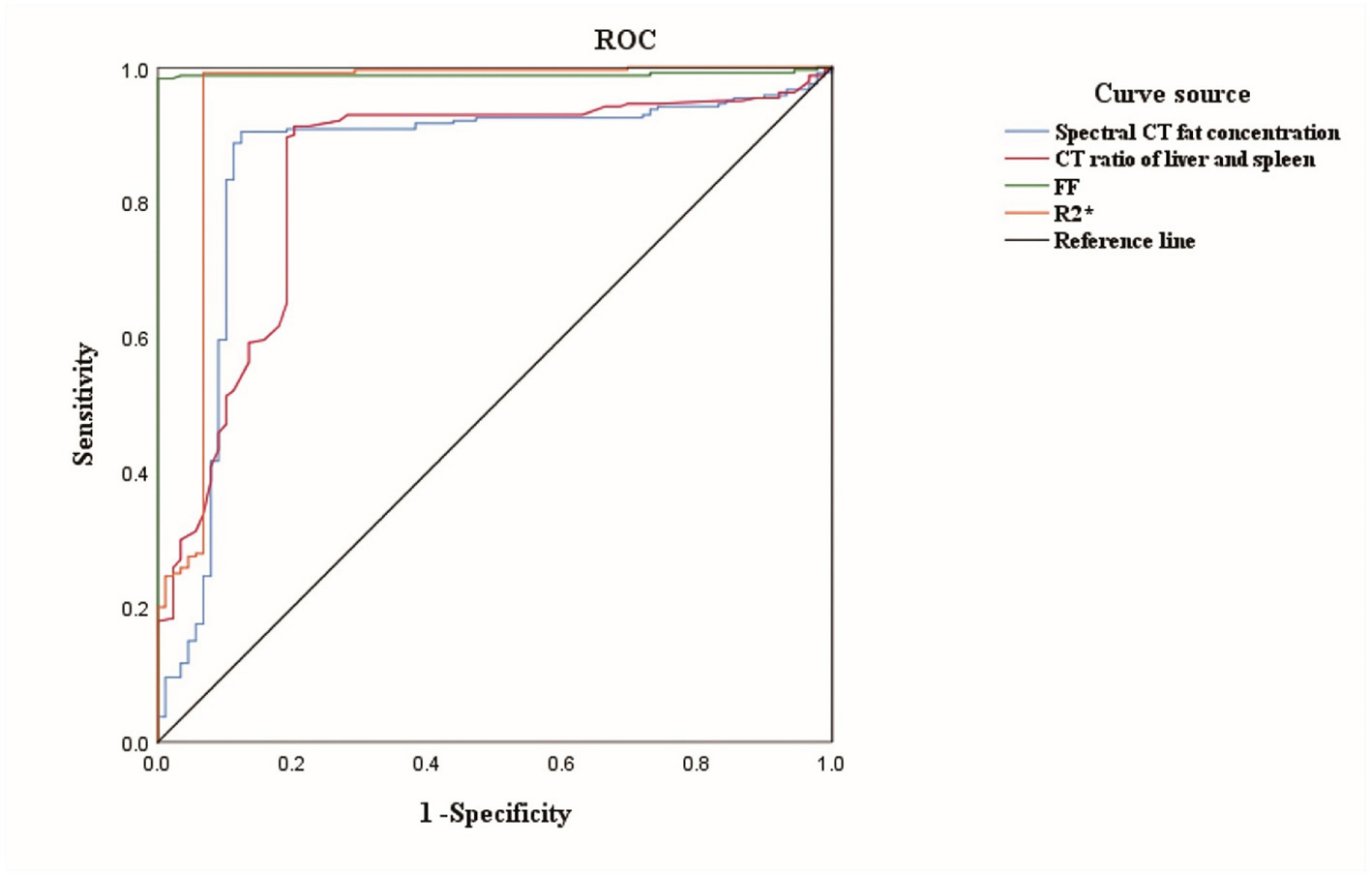
ROC (receiver operating characteristic) curves comparing CT (computed tomography) parameters and FF (fat fraction) for diagnosing MASLD (metabolic dysfunction–associated steatotic liver disease).

All 329 subjects were randomly divided into a training set (*n* = 230; 168 MASLD, 62 CG) and a validation set (*n* = 99; 72 MASLD, 27 CG) in a 7:3 ratio. In ROC analysis, MRI fat fraction (FF) showed the highest diagnostic performance for MASLD (AUC = 0.89, 95% CI: 0.84–0.93), followed by R2^*^ (AUC = 0.84, 95% CI: 0.78–0.90), CT fat concentration (AUC = 0.80, 95% CI: 0.73–0.86), and the liver-to-spleen attenuation ratio (AUC = 0.76, 95% CI: 0.69–0.83) (Table 5, Figure 6). Among these imaging parameters, MRI-derived FF showed the strongest ability to distinguish MASLD from controls. R2^*^ and CT fat concentration also showed good diagnostic performance, whereas the liver-to-spleen ratio showed relatively lower performance. These findings indicate that MRI-derived parameters, particularly FF and R2^*^, performed better than CT-based parameters for identifying MASLD.

**Table 5:** Comparison of ROC curve AUC between CT and MRI fat quantitative parameters in the diagnosis of MASLD.

Index	*Z* value	*P* value
Energy spectrum CT fat concentration vs. liver-to-spleen CT ratio	0.220	0.826
Energy spectrum CT fat concentration vs. FF	0.999	< 0.001
Energy spectrum CT fat concentration vs. R2^*^	0.998	0.002
CT ratio of liver and spleen vs. FF	0.999	0.001
Liver-to-spleen CT ratio vs. R2^*^	0.987	0.013
FF vs. R2^*^	0.950	0.050

CT, computed tomography; FF, fat fraction.

### 3.6 Analysis of diagnostic performance of training set

ROC analysis in the training set showed that FF had the largest AUC for diagnosing MASLD, followed by R2^*^, as shown in Table 1 and Figure 6. The diagnostic performance of energy spectrum CT fat concentration and the liver-spleen CT ratio was comparable, with no significant difference between them (P > 0.05). Both CT-based parameters showed lower diagnostic performance than FF and R2^*^ (*P* < 0.05). No significant difference was found between FF and R2^*^ in diagnosing MASLD (*P* > 0.05, Table 5).

### 3.7 Validation set performance verification

The diagnostic performance of CT and MRI fat quantification parameters was evaluated in the validation cohort (*n* = 99). Among all parameters, MRI-derived FF demonstrated the highest diagnostic performance, with a specificity of 97.9%, sensitivity of 98.7%, and accuracy of 98.5%. R2^*^ showed the second-best performance, with a specificity of 96.4%, sensitivity of 91.7%, and accuracy of 93.0%. CT-based parameters, including energy spectrum fat concentration and liver-spleen ratio, also showed acceptable diagnostic performance, although their sensitivity, specificity, and overall accuracy were lower than those of the MRI-derived parameters (Table 2). The corresponding AUCs in the validation set were 0.855 for CT fat concentration, 0.843 for the liver-spleen ratio, 0.989 for FF, and 0.946 for R2^*^, indicating consistent diagnostic performance across cohort.

### 3.8 Clarification of FF values in MASLD group

The mean FF value of 52.65% in the MASLD group may reflect the inclusion of patients with marked or advanced steatosis. Although this value appears relatively high, it is plausible in patients with severe hepatic fat accumulation. In the revised manuscript, the interpretation of this finding has been clarified to indicate that the reported mean FF may have been influenced by cases with more advanced steatosis, whereas milder cases of MASLD may show lower FF values in routine clinical practice.

## 4 Discussion

Our study demonstrated that MRI-derived FF provided the highest diagnostic performance for detecting MASLD, followed by R2^*^, whereas CT-based measures, including fat concentration and liver-to-spleen (L/S) ratio, showed comparatively lower performance. These findings suggest that MRI-based fat quantification may have greater value than CT-based techniques for evaluating hepatic steatosis.

This observation is consistent with previous studies demonstrating the high sensitivity and reproducibility of MRI for hepatic fat quantification. For example, Reeder et al. and Starekova et al. reported that MRI proton density fat fraction (PDFF) provides accurate, and reproducible quantification of hepatic fat content and correlates strongly with histologic steatosis grades (28–30). Similarly, Jung et al. (20) demonstrated that MRI-based techniques outperform quantitative ultrasound for detecting steatosis in patients with suspected MASLD. Our findings further suggest that FACT-derived FF shows promising performance and may be a useful alternative when MRI-PDFF is unavailable or when concurrent R2^*^ assessment is clinically relevant.

### 4.1 Comparison with other MRI fat-quantification techniques and generalizability

The FACT approach used in this study is conceptually similar to other chemical shift–encoded (multi-echo Dixon) MRI techniques that estimate proton density fat fraction (PDFF) and R2^*^, such as IDEAL-IQ and mDIXON. Prior work has shown that confounder-corrected Dixon/IDEAL-type methods can provide robust PDFF and T2^*^/R2^*^ estimates when appropriate modeling is applied, (including multi-peak fat spectrum and T2^*^ correction), and IDEAL-IQ has been reported to be relatively stable even when R2^*^ changes substantially (31). Cross-vendor standardization studies also support good reproducibility of PDFF and R2^*^ across vendors and field strengths when using harmonized acquisition and fitting strategies, including standardized IDEAL-based implementations and multi-center/multi-vendor phantom validations (32, 33). In addition, vendor-neutral PDFF processing approaches have shown strong correlation with histology across different MR devices, supporting the broader applicability of PDFF-based biomarkers beyond a single vendor pipeline. Nevertheless, absolute PDFF/R2^*^ values can still vary with scanner hardware, sequence details (34), reconstruction methods, and fitting algorithms. Therefore, our thresholds and performance metrics should be interpreted in the context of our vendor-specific FACT implementation, and external validation on other platforms, such as IDEAL-IQ and mDIXON, is warranted. Future multicenter studies using standardized acquisition protocols or vendor-neutral PDFF processing would further improve generalizability.

Importantly, we observed significant positive correlations between FACT-derived parameters (particularly FF and R2^*^) and metabolic biomarkers such as AST, TC, and TG, suggesting that these imaging parameters may not only quantify hepatic fat but also reflect metabolic alterations associated with MASLD. In contrast, the lack of strong associations with inflammatory cytokines implies that FACT-derived metrics may primarily represent fat accumulation rather than inflammatory activity.

The inclusion of R2^*^analysis offers an added advantage. Elevated hepatic iron deposition, reflected by R2^*^, frequently coexists with MASLD and contributes to oxidative stress, inflammation, and fibrosis (35–37). Moreover, hepatic iron concentration has been shown to correlate with metabolic abnormalities such as insulin sensitivity in non-alcoholic fatty liver disease, supporting the clinical relevance of evaluating iron alongside fat in MASLD (38). Therefore, simultaneous assessment of fat and iron using FACT MRI may provide a more comprehensive evaluation of hepatic pathology and improve risk stratification in patients with MASLD.

A novel aspect of our study is the integrated comparison of imaging-derived fat parameters with liver-related serum markers within the same cohort. While imaging and serological markers are often evaluated separately, our results suggest that FACT-based MRI parameters are associated with biochemical indicators of liver injury and lipid metabolism. This supports the potential role of FACT MRI as a complementary tool for both morphologic and biochemical assessment in MASLD.

Another strength of our study is the use of both training and independent validation cohorts, which enhances the robustness and reproducibility of our findings. The optimal cut-off values for FF and R2^*^ identified in the training cohort maintained their diagnostic performance in the validation cohort, supporting their potential integration into clinical workflows using standardized thresholds.

### 4.2 Clarification of diagnostic performance

Diagnostic performance should be interpreted cautiously. Given the lack of uniform gold standard validation across the entire cohort, the findings of this study should be considered exploratory. Although the FACT-derived parameters showed promising results, their apparent diagnostic performance requires further confirmation using more rigorous reference standards, such as MRI-PDFF or liver biopsy. In addition, only 132 of the 329 participants underwent liver biopsy, whereas the remaining participants were diagnosed using ultrasound combined with clinical criteria. Because ultrasound has limited sensitivity for mild steatosis, disease severity may have been underestimated in some patients, particularly those with early or mild disease. As a result, the use of ultrasound-based diagnoses may have introduced incorporation bias and may have inflated the observed diagnostic performance of the imaging biomarkers. Therefore, future studies using MRI-PDFF or histopathology as the reference standard are needed to validate these findings more definitively.

### 4.3 Discussion of ultrasound techniques and study focus

Although these methods have shown promise for the non-invasive assessment of hepatic steatosis, they remain operator-dependent and may be less accurate in patients with obesity or heterogeneous fat distribution. In addition, the lack of standardized thresholds and validated reference standards limits their broader clinical adoption. In contrast, CT and MRI fat quantification, particularly the FACT sequence, offer a more reproducible and established approach for evaluating MASLD. Therefore, we focused on CT and MRI to provide a direct comparison of imaging biomarkers with potential clinical utility in MASLD.

### 4.4 Novelty and clinical implications

A key strength of this study is the direct head-to-head comparison of FACT-derived MRI parameters and energy spectrum CT indices within the same patient cohort. While previous studies have primarily focused on validating MRI PDFF or ultrasound-based techniques, few investigations have simultaneously evaluated FACT-derived FF and R2^*^alongside CT-derived metrics and liver-related serum biomarkers.

An interesting observation was the positive correlation between R2^*^ and systemic lipid markers (TC and TG), suggesting that hepatic iron deposition may be linked to lipid metabolism. This finding highlights the potential of R2^*^ as not only an iron quantification parameter but also a marker that may indirectly reflect metabolic dysfunction.

Clinically, these findings emphasize the potential of FACT MRI as a comprehensive, non-invasive diagnostic tool capable of assessing both hepatic fat and iron within a single protocol. This dual assessment may support improved risk stratification and individualized patient management in MASLD, especially in cases where MRI-PDFF is unavailable or when concurrent iron overload is suspected.

Despite these strengths, this study has several limitations. First, the use of ultrasound-based diagnoses in part of the cohort may have introduced incorporation bias, which could have led to overestimation of diagnostic performance. Future studies using MRI-PDFF or histopathology as the reference standard are needed to validate the imaging biomarkers more accurately.

Second, the retrospective design may have introduced selection bias and limits causal inference. Third, quantitative histologic scoring, such as detailed NAS or fibrosis staging, was not available for correlation with imaging severity. This was mainly due to the retrospective nature of the study and the lack of standardized archival histopathologic scoring data. Re-examining all biopsy specimens was not feasible within the scope of this study. Fourth, this study focused on diagnosis rather than longitudinal monitoring; therefore, future studies should evaluate the value of FACT in tracking disease progression and treatment response. Fifth, Fifth, the generalizability of our findings should be confirmed in multicenter and multiethnic populations with varying degrees of steatosis and fibrosis. Overall, the absence of a uniform histologic reference or MRI-PDFF-based validation may limit the generalizability of the findings, and future prospective studies are warranted.

## 5 Conclusion

In conclusion, this study suggests that FACT-derived MRI parameters, particularly FF and R2^*^, may perform better than CT-based indices for the non-invasive evaluation of MASLD. These parameters also showed correlations with metabolic and liver injury biomarkers, indicating their potential value in integrated disease assessment. By comparing FACT-derived MRI parameters with CT-based indices and serum biomarkers in the same cohort, this study supports the potential role of FACT MRI in the comprehensive assessment of hepatic steatosis. The ability to simultaneously characterize hepatic fat and iron content further highlights its possible clinical utility. However, because of the exploratory nature of the study, the partial use of ultrasound-based diagnoses, and the absence of uniform validation with liver biopsy or MRI-PDFF, the findings should be interpreted with caution. Future prospective studies using validated reference standards are needed to confirm the diagnostic value and clinical applicability of CT- and MRI-derived fat quantification in MASLD.

## Data Availability

The raw data supporting the conclusions of this article will be made available by the authors, without undue reservation.
